# Left-Deviating Prism Adaptation in Left Neglect Patient: Reflexions on a Negative Result

**DOI:** 10.1155/2012/718604

**Published:** 2012-09-25

**Authors:** Jacques Luauté, Sophie Jacquin-Courtois, Jacinta O'Shea, Laure Christophe, Gilles Rode, Dominique Boisson, Yves Rossetti

**Affiliations:** ^1^Pole de rééducation et réadaptation, Plateforme mouvement et handicap, Hôpital Henry Gabrielle, Hospices Civils de Lyon, 69000 Lyon, France; ^2^ImpAct, INSERM (U1028), CNRS (UMR5292), Lyon Neuroscience Research Center, 69000 Lyon, France; ^3^Université de Lyon, Université Lyon 1, F-69100, Villeurbanne, France; ^4^Oxford Centre for Functional MRI of the Brain, Nuffield Department of Clinical Neurosciences, University of Oxford, Oxford OX3 9DU, UK

## Abstract

Adaptation to right-deviating prisms is a promising intervention for the rehabilitation of patients with left spatial neglect. In order to test the lateral specificity of prism adaptation on left neglect, the present study evaluated the effect of left-deviating prism on straight-ahead pointing movements and on several classical neuropsychological tests in a group of five right brain-damaged patients with left spatial neglect. A group of healthy subjects was also included for comparison purposes. After a single session of exposing simple manual pointing to left-deviating prisms, contrary to healthy controls, none of the patients showed a reliable change of the straight-ahead pointing movement in the dark. No significant modification of attentional paper-and-pencil tasks was either observed immediately or 2 hours after prism adaptation. These results suggest that the therapeutic effect of prism adaptation on left spatial neglect relies on a specific lateralized mechanism. Evidence for a directional effect for prism adaptation both in terms of the side of the visuomanual adaptation and therefore possibly in terms of the side of brain affected by the stimulation is discussed.

## 1. Introduction

Patients with right cerebral hemisphere lesions often show a reduced tendency to respond to stimuli and to search actively for them in the contralateral part of space [1]. This condition described as left spatial neglect is typically demonstrated by clinical observation and simple perceptual motor tests such as a line bisection or cancellation test [2]. Left spatial neglect occurs in about 25–30% of all stroke patients [3], and although some degrees of spontaneous recovery occurs [4], the disorder persists chronically in many cases [5]. Frequently associated with contralesional motor or somatosensory deficit, left spatial neglect is recognized as one of the main factors associated with poor functional outcome [6–8]. For these reasons, the improvement of left spatial neglect over and above spontaneous recovery represents a challenge in the area of neurological rehabilitation. Over the past 60 years, many different attempts to alleviate this impairment have been developed (for a review see [9]).

Among these, prism adaptation is one of the most promising therapeutic interventions [10]. Prism adaptation has been widely used since the end of the nineteenth century as a paradigm to demonstrate visuomotor short-term plasticity [11]. Exposure to prisms produces a lateral shift of the visual field so that the visual target appears at a displaced position. Adaptation to such an optical induced shift critically requires a set of successive perceptual-motor pointing movements. While the initial movements tend to approximate to the virtual position of the target, subsequent pointing movements ensure that the pointing error rapidly decreases so that subjects can readily point towards the real target position [12]. This initial error reduction comprises a “strategic component” of the reaction to prisms and does not necessarily produce adaptation at this stage [13]. To obtain robust compensatory after-effects following removal of prisms, further pointing movements are required. These reinforce the sensory-motor adaptation and are considered characteristic of the “real” or “true” adaptive component of the adaptation (e.g., [14]). After-effects result from a compensatory shift in manual straightahead pointing in a direction opposite to the original visual shift produced by prisms. Rossetti et al. [15] proposed that the adaptation to right-deviating prisms with leftward compensatory after-effects (using the intact right hand) improved left neglect symptoms. In this study, a significant improvement was demonstrated across a variety of different standard paper and pencil tests (line bisection, line cancellation, copying a scene, and reading a simple text). Subsequent studies have shown that these clinical effects could extend to numerous neglect-related processes (for a review see [16]) such as straightahead pointing [17], visual exploration toward the left hemispace [18], postural control [19], contralesional somatosensory perception [20–22], temporal order judgment [23], and mental representation [24–26]. From a rehabilitation perspective, the long-term beneficial effect on several functional measures set this intervention apart from the other attempts. (i) Farné et al. [27] found a reduction of spatial dyslexia still present one day after a single session of prism adaptation in a group of 6 patients with left spatial neglect. (ii) Rode et al. [28] reported a positive effect of prism adaptation on spatial dysgraphia. The improvement concerned the right-page preference and was maintained up to 4 days after a single session of prism adaptation. (iii) Jacquin-Courtois et al. [29] and Watanabe and Amimoto [30] reported an improvement of wheelchair navigation after a single session of prism adaptation in two single-case studies. (iv) A long-lasting amelioration, up to five weeks, was reported on several functional tasks following a twice-daily adaptation program during a period of two weeks [31, 32] or one-daily prism-adaptation session during two weeks [33, 34].

This impressive generalization and long-standing effects of prism adaptation have revived interest in the neuro-cognitive mechanisms by which it has been achieved. The most two basic questions about the mechanisms of action of prism adaptation are (i) whether adaptation per se is necessary to produce cognitive after-effects or whether simple visuomanual pointing could produce similar effects, and (ii) whether this adaptation is specific in terms of its direction. As a matter of facts, such specificity has been demonstrated in healthy individuals (see for review [35]), in patients with complex regional pain syndrome [36], but no data is available on neglect patients. The purpose of the present study was to evaluate the directional specificity of prism adaptation in neglect patients. Given that the effect of right-deviating prisms on left spatial neglect is already well documented (cf. supra), this work was designed to evaluate the effect of left-deviating prisms on left spatial neglect. Since it has been shown that adaptation to right-deviating prism may affect differently straightahead pointing movements and attentional tasks [17], the effect of left-deviating prism was measured both on straightahead pointing movements and several attentional tasks classically used to assess left spatial neglect.

## 2. Methods

### 2.1. Participants

Patients were selected from the Neuro-rehabilitation Department of the Hospices Civils de Lyon, France. Inclusion criteria were right-handed patients with left spatial neglect after right hemispheric ischemic or hemorrhagic stroke. Patients with previous history of stroke, psychiatric diseases, global cognitive deterioration, or any impairment that could compromise comprehension and compliance with the tasks were excluded.

For all patients screened, hand preference was assessed by the Edinburgh inventory [37]. Left spatial neglect was assessed using a battery of six paper and pencil tests: line cancellation, balloon test, line bisection, copy of a scene, drawing from memory, simple text reading (cf.  Section 2.2.2. for description). The presence of hemianopia was assessed by means of Goldman perimetry. A cerebral computerized tomography (CT) or MRI scan was performed for each patient in order to specify the type of lesion (ischemic or hemorrhagic), to rule out any other relevant prestroke lesions and to determine the anatomic location of the lesion.

A group of healthy subjects was included for comparison purposes.

This study was conducted with the informed consent of the participants, in agreement with the French law (March 2002) and the Helsinki declaration relative to patient's rights.

The sample comprised six healthy subjects and five patients aged between 67 and 80 years old (see Table 1 for clinical profiles of each patient). The mean time period between stroke onset and inclusion was 1.5 months (range: 1 to 2.5 months). One patient had a hemorrhagic stroke (patient 2); the four others had an ischemic stroke: two in the posterior part of the superficial middle cerebral artery territory (patients 1 and 3), one in the anterior part of the superficial middle cerebral artery territory (patient 5), and one in the deep part of the middle cerebral artery territory (patient 4).

**Figure 1:**
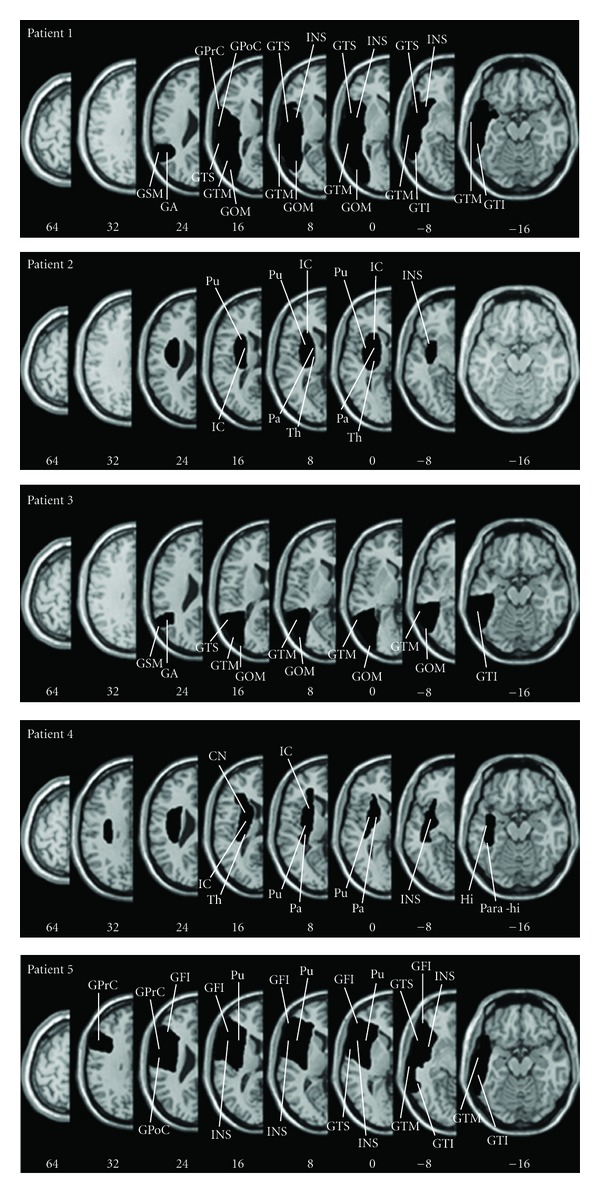
Lesion anatomy. For each patient, all lesions were mapped using the free MRIcro software and were drawn manually on slices of the high-resolution 3D T1-weighted template MRI scan. This template is oriented to match the Talairach space. Lesions were mapped onto the horizontal slices that correspond to Z-coordinates −16, −8, 0, 8, 16, 24, 32, 64 in the Talairach space by using the identical or the closest matching horizontal slices of each individual. Following radiological convention, the right cerebral hemisphere is displayed on the left side. *Abbreviations*: CN, caudate nucleus; GFI, gyrus frontalis inferior; GOM, gyrus occipitalis medius; GPrC, gyrus precentralis; GPoC, gyrus postcentralis; GTM, Gyrus temporalis medius; GTI, gyrus temporalis inferior, GTS, gyrus temporalis superior; Hi, hippocampus; IC, internal capsule; INS, insula; Pa, pallidum; Para-hi, parahippocampus; Pu, putamen; Th, thalamus; GSM, gyrus supra-marginalis; GA, gyrus angular.

Lesion analysis showed the involvement of the inferior, middle, and superior temporal gyri in three patients (patient 1, patient 3, and patient 5); the temporoparietooccipital junction was damaged in two patients (patient 1 and patient 3). Lesions of other brain structures involved the somatosensory parietal cortex (patient 1 and patient 5), the primary motor cortex (patient 1 and patient 5), the occipital cortex (patient 1 and patient 3), the prefrontal and the orbito-frontal cortex (patient 5), the insula (patient 1, patient 2, patient 4, and patient 5), the thalamus (patient 2 and patient 4), the putamen and pallidum (patient 2, patient 4, and patient 5), the internal capsule (patient 2 and patient 4), the caudate nucleus, the hippocampus, and parahippocampus (patient 4).  Figure 1 shows selected horizontal sections of the lesions for each patient.

### 2.2. Experimental Procedure

Patients' performance was investigated in sessions that took place before prism adaptation (referred to as “pre”), immediately after (post), and 2 hours after (late). Healthy subjects performed the same tasks as patients before (pre) and immediately after (post) prism adaptation. During each session, left spatial neglect was assessed using line cancellation, balloon test, line bisection, copy of a scene, drawing from memory, and a simple text reading (cf.  Section 2.2.2. for description). In order to check whether healthy subjects and patients correctly adapted to prisms, a measure of straightahead pointing was performed before adaptation (pre) and after participants had completed the immediate neuropsychological tests (post) as in Rossetti et al. [15].

#### 2.2.1. Straight-Ahead Pointing

The participant was seated blindfolded in front of a horizontal box that allowed for an electronic measurement of the finger movement endpoints with an accuracy of 1 deg. Participants were required to point straightahead while their head was kept aligned with the body's sagittal axis. Seven pointing trials were performed during each of the two assessments.

#### 2.2.2. Assessment of Left Spatial Neglect. Test Details

**Line Cancellation [38]**

This test consists of an A4 page containing 40 lines arranged in different direction. The page is placed at body midline. Participants were instructed to cross out all the lines on the page. The score was the total number of lines crossed.

**The Balloon Test [39]**

This test consists of two subtests, carried out on two A3 landscape-orientated stimulus sheets, each containing 202 items (circles or balloons). In the first subtest “pop-out”, 22 target balloons are interspersed between 180 circles which play the role of distractor. Subjects were asked to cross out as many balloons as they could find. This test is based on the phenomenon of perceptual “po-pout,” that is, the time taken to detect target of this kind does not increase significantly as the number of distractors increase [40]. In the second subtest “search,” the number and position of the balloons and circles are exactly the reverse; thus 22 of the 202 items are circles to be cancelled and the other 180 items are balloons. In this test, subjects were required to cancel out as many circles as they could find. In this test, the targets do not “pop out.” Rather they have to be searched, and therefore, this test requires a greater demand on attention. In both subtests, the score represents the number of targets correctly cancelled.

**Line Bisection [41]**

Participants were presented with an A4 page, in front of their body midline, containing twenty lines of different length ranging from 100 mm to 200 mm. Participants were instructed to cut each line in half by placing a small pencil mark through each line as close to its center as possible. The score was the mean percentage of deviation from the true center of the line (the score is positive when the deviation is in the right direction and negative when the deviation is in the left direction).

**Copy of a Scene [42]**

Participants were required to reproduce a picture made up of five items (4 trees and a house) in the space bellow it. Performances were assessed by two scores: (i) the number of items reproduced and (ii) the number of items symmetrically depicted.

**Drawing from Memory**

Participants were simply asked to draw a daisy without any model. A score of 1 was given when the daisy was highly asymmetrical, 2 when the drawing was moderately asymmetrical, and 3 when the drawing was symmetrical.

**Simple Text Reading**

Patients were required to read a simple text. The score on this test represented the number of words omitted or modified.

#### 2.2.3. Prism Adaptation (See Figure 2)

The adaptation procedure involved the participants having to wear prismatic goggles that produced a 10° leftward shift of the visual wide-field that is in the opposite direction to Rossetti et al. (1998) [15]. While wearing prisms, the participant was required to make—as fast as possible—a series of approximately 50 pointing responses, with his/her right hand, to visual targets located to the left and right side of midline. The procedure lasted approximately five minutes. In order to ensure optimal adaptation, visual feedback of the starting point of the hand was always occluded and the pointing trajectories were visible.

#### 2.2.4. Statistical Analysis

The first analysis was to test whether healthy subjects and patients correctly adapted to left-deviating prisms. We carried out *t*-tests to compare the average end-position before (pre) and after (post) prism adaptation.

In order to evaluate the presence of an amelioration of left neglect symptoms after prism adaptation, an analysis of variance with repeated measure (ANOVA) was performed on each neuropsychological test, using sessions (pre, post, late) as factor. Hence, for a specified test, the null hypothesis (*P* value >0.05) is the absence of difference between sessions of the mean score across patients. The alternative hypothesis (*P* value <0.05) can be written as follow: at least one of the mean score differs between sessions. In this latter case, a post hoc Sheffé test was carried out in order to compare the mean scores across sessions: “pre versus post,” “pre versus late,” and “post versus late.”

## 3. Results

For the healthy controls, a significant displacement of the straightahead pointings to the right was observed after exposure to left deviating prisms without significant modification of the performances on the attentional paper and pencil tests.

For the neglect patients, no significant effect of prism exposure was observed neither on the straight-ahead pointing task nor on the neuropsychological tests.

### 3.1. Straight-Ahead Pointing

**Figure 3:**
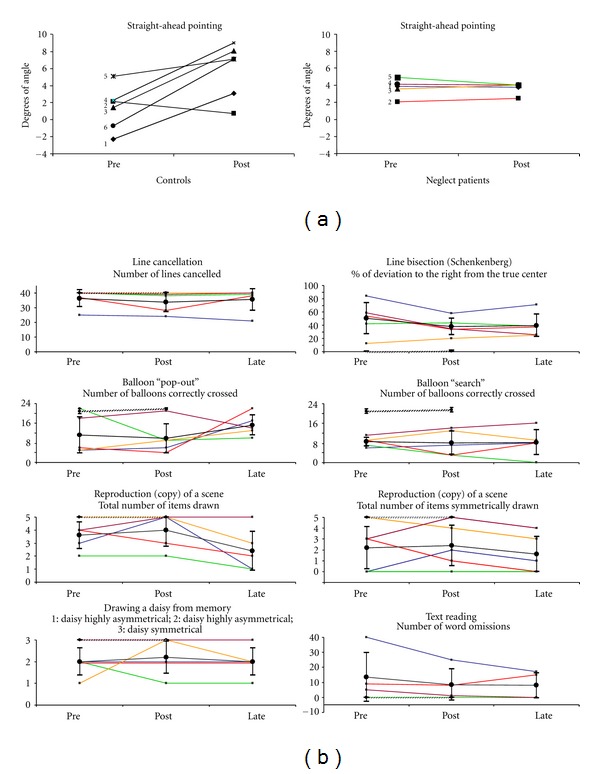
Straighthead pointing movements before and after prism adaptation for healthy controls (left) and neglect patients (right). (a) For each subject, the average end-position of straightahead pointing movements is represented before (pre) and after (post) left-deviating prism adaptation. Deviation from body midline is displayed in degrees of angle in positive value for right deviation and negative value for left deviation. Numbers refer to patient's identification (cf. Table 1) with the following color code: patient 1 (blue); patient 2 (red); patient 3 (orange); patient 4 (purple); patient 5 (green). (b) Left spatial neglect assessment before (pre), immediately after (post), and 2 hours after (late) prism adaptation. For each test, the graph represents the mean score ±95% confident interval for the group of five patients at each session. Individual curves are represented using the same color code as in Figure 3(a). Moreover, performances of the healthy controls (95% confident interval) are displayed in cross-hatching. For tests and scores description see Section 2.2.2.

Controls. Before left-deviating prism adaptation (pre), the group analysis showed that the mean end-position of 7 straightahead pointing trials was shifted 1.3 degrees to the right of the body midline (range: −2.3° to 5.1°). After prism adaptation (post), the mean deviation was significantly displaced to the right (mean position after prism adaptation: 5.8 degrees to the right of the body midline; range: 0.7° to 9.0°). Comparison between trials performed before and after prism adaptation was significant (*t* = 3.15; *P* = 0.026). (cf.  Figure 3(a) left graph).

Patients. Before left-deviating prism adaptation (pre), the group analysis showed that the mean end-position of 7 straightahead pointing trials was shifted 3.7 degrees to the right of the body midline (range: 2.0° to 4.9°). After prism adaptation (post), the mean end-position was unchanged (mean: 3.7 degrees to the right; range: 2.4° to 4°). Comparison between trials performed before and after prism adaptation was not significant (*t* = 0.74; *P* = 0.48). Individually, the difference of end-position before and after prism adaptation was always less than 1 degree of angle (cf.  Figure 3(a) right graph).

### 3.2. Effect of Adaptation to Left-Deviating Prism on Left Spatial Neglect (Figure 3)

None of the paper and pencil attentional tests have been significantly modified by left-deviating prisms in the control group. The 95% confident interval of healthy subjects' performances is displayed on Figure 3(b) for each test.

For the neglect group, numerical results are reported for each test in the following section and in Figure 3(b).

#### 3.2.1. Line Cancellation

An average of 36.4 lines were cancelled before prism adaptation, 33.8 immediately after prism adaptation, and 35.6 two hours later (standard error of mean = 3.3). Analysis of variance showed no significant difference between sessions, *F* (2, 8) = 1.31; *P* = 0.32.

#### 3.2.2. The Balloon Test

In the “pop-out” subtest, a mean of 11.2 targets balloons were crossed before prism adaptation, 9.8 immediately after, and 15.2 two hours later (standard error of mean = 3.0). Analysis of variance showed no significant difference between sessions *F* (2, 8) = 0.86; *P* = 0.46.

In the “search” subtest, a mean of 8.4 circles were crossed before prism adaptation, 8.0 immediately after prism adaptation, and 8.2 two hours later (standard error of mean = 2.1). Analysis of variance showed no significant difference between sessions *F* (2, 8) = 0.02; *P* = 0.98.

#### 3.2.3. Line Bisection

Before prism adaptation, patients bisected lines with a mean deviation calculated at 50.3 percent on the right of the true centre; this deviation was 35.8 immediately after prism adaptation and 39.5 two hours later (standard error of mean = 9.0). Analysis of variance showed no significant difference between sessions *F* (2, 8) = 2.27; *P* = 0.17.

#### 3.2.4. Copy of a Scene

Before prism adaptation, an average of 3.6 of the five items was copied and an average of 2.2 items was symmetrically copied. Immediately after prism adaptation, an average of 4 items was copied and an average of 2.4 items was symmetrically copied. Two hours after prism adaptation, an average of 2.4 items was copied and an average of 1.6 items was symmetrically copied. Standard error of mean was 0.64 for the total number of items copied and 0.91 for items symmetrically copied. Analysis of variance showed no significant difference between sessions both for the total number of items copied *F* (2, 8) = 3.92; *P* = 0.06 and for the number of items symmetrically copied *F* (2, 8) = 0.78; *P* = 0.49.

#### 3.2.5. Drawing from Memory

The daisy was moderately asymmetrical before prism adaptation (mean = 2), immediately after (mean = 2.2), and two hours later (mean = 2). Standard error of mean for this test was 0.34. Analysis of variance showed no significant difference between sessions *F* (2, 8) = 2.21; *P* = 0.81.

#### 3.2.6. Simple Text Reading

Before prism adaptation, an average of 13.5 words were omitted, 8.5 immediately after prism adaptation, and 8.0 two hours later (standard error of mean = 6.7). Analysis of variance showed no significant difference between sessions *F* (2, 8) = 0.92; *P* = 0.45.

## 4. Discussion

The present study showed that patients with left spatial neglect are not affected by prism adaptation to a leftward optical shift. Indeed, neither the rightward deviation of straightahead pointing nor left spatial neglect, as assessed by a battery of classical paper and pencil tests, has been significantly improved or modified after a single session of visuomotor adaptation to left-deviating prisms. Not only do these results suggest that there is a directional specificity of the prisms, but they also show that no cognitive effects are found in the absence of adaptation. The present results play against the hypothesis that active exposure to a simple modification of sensori-motor coordinates is sufficient to reduce left spatial neglect. The short duration of the adaptation procedure cannot explain independently the absence of sensorimotor after-effects given that healthy controls adapt to prisms with the same procedure and neglect patients show sensori-motor after-effects, even larger than controls, when exposed to right-deviating prisms during the same amount of time [15].

### 4.1. Specific Directional Effects of Prism Adaptation in Neglect Patients

#### 4.1.1. Adaptability to Wedge Prisms

In our experiment, none of the 5 neglect patients showed a consistent sensori-motor adaptation to left-deviating prisms. A similar result was already reported in experiment 1 of the original research performed by Rossetti et al. [15]. In this latter study, eight patients with left spatial neglect were randomly assigned to a session of left- or right-deviating prism adaptation. Adaptability was assessed by measuring body-midline demonstration (i.e. straightahead pointing in the dark). In contrast to normal subjects, results showed that patients with left neglect adapted only to right-deviating prism and not to left-deviating prism. The effect of left-deviating prism adaptation on left spatial neglect symptoms was not specifically assessed in this latter work.

These results suggest that patients with left spatial neglect after right-brain damage are not able to adapt to left-deviating prisms whereas they are able to adapt to right-deviating prisms. This result contrasts with the finding of Weiner et al. [14] that the only lesion site that impaired prism adaptation was within the cerebellum (see [43]). Although Weiner et al. [14] tested groups of patients with left versus right hemisphere lesion, no information is provided concerning the assessment of left spatial neglect. In their study it was stated that only patients with occipital lesion exhibited reduced negative after-effects. However in our group, the lesion overlapped the occipital cortex in only two out of the five patients. One explanation to this intriguing negative result could be related to the absence of detection of the visual errors by left neglect patients in the case of left-deviating prisms. Indeed, the first pointing movements with left-deviating prisms are shifted to the left side of the visual target, and considering that patients focus their vision on the target position, the visual error lies in the left visual field. Hence, it is not surprising that this visual error, which represents the first necessary signal for prism adaptation, is not even implicitly detected in patients with left spatial neglect.

Alternatively, it is possible that visual realignment after leftward-deviating prisms critically requires the integrity of the right hemisphere in contrast to visual realignment after rightward deviating prisms. As regard to this hypothesis, it is interesting to consider the directional asymmetry for visual after-effects observed in healthy subjects after a visual adaptation to leftward versus rightward prism displacement [44]. This could explain why right-brain-lesioned patients are only able to adapt to rightward deviating prisms. The larger amplitude of sensori-motor after-effects observed in right brain damaged neglect patients compared to healthy controls [15] is another interesting issue which could be related to the asymmetrical integration of the prism adaptation process.

#### 4.1.2. Effect on Left Spatial Neglect and Related Symptoms

Our results showed for the first time that left-deviating prisms had no effect on various symptoms of left spatial neglect. Previous studies have reported a similar lateralized specificity of prism adaptation on several neglect-related symptoms. Tilikete et al. [19] investigated the effect of prism adaptation on postural imbalance in a group of 15 left hemiparetic patients, randomly exposed to right-deviating prism, left-deviating prism, or neutral goggles. The lateral displacement of the centre of pressure observed in the pretest was significantly reduced specifically following right-deviating prisms. Finally, for one of the neglect patients (patient 4) included in the experiment performed by Maravita et al. [21], contralesional tactile perception and visual extinction were improved only after adaptation to right-deviating prism and not after adaptation to left-deviating prism.

Hence, these results favour a specificity of prism adaptation in terms of the direction of optical shift: only adaptation to right-deviating prisms can improve left spatial neglect. The most obvious explanation to account for these results is related to the absence of adaptability to left-deviating prism for patients with left spatial neglect (cf.  Section 4.1.1). These results support the hypothesis that the presence of sensori-motor after-effect is a necessary condition to influence the highest cognitive levels of space and action representation subserving neglect recovery. However, as pointed out by Rode et al. [16], several studies have shown that the quantitative relationship between the amplitude of after-effect and neglect amelioration is not obvious (e.g., [45]).

### 4.2. Specific Directional Cognitive Effects of Prism Adaptation in Healthy Subjects

Interestingly, the cognitive effects of prism, in non-brain-damaged subjects, are also supported by an asymmetrical pattern of performance. Colent et al. [46] examined the possibility that visuomotor adaptation to left- or right-deviating prisms could generate a bias on a line bisection task. Only adaptation to left-deviating prisms induced a rightward bias on the perceptual version of the line bisection task. This result was then confirmed by Berberovic et al. [47]. Michel et al. [48] investigated the effect of prism adaptation on postural control in healthy subjects. Fourteen participants were either adapted to a leftward or rightward visual shift and it was found again that only adaptation to a leftward visual shift induced significant rightward postural bias. (For a review see [35]. In another experiment, Michel et al. [49] showed asymmetric effects after manual or locomotor adaptation (walking along a rectangle drawn on the floor with prismatic google) to a leftward or ritghtward optical deviation on a goal-oriented locomotor task (estimation of the spatial location of a visual target with body displacement). On the goal-oriented locomotor task which comprises a spatial dimension, the rightward after-effects generated by left-deviating prisms were greater than the leftward after-effects generated by right-deviating prisms. This result suggests that in contrast to rightward-deviating prisms generating only sensori-motor adaptation, leftward-deviating prisms may induce both sensori-motor and an additional cognitive after-effect.

Striemer et al. [50] investigated whether prism adaptation could influence visual attention, as assessed by a visual attention cueing paradigm. Two versions of the task were employed depending on the delay separating cue onset and target onset. In the reflexive version, the delay was short (50 to 300 ms), whereas it was longer in the voluntary version (300 to 500 ms). Healthy participants were divided in three groups: left-deviating prisms, right-deviating prisms, and neutral goggles. The main result was an increase of voluntary attention efficiency for both left and right visual space after adaptation to left-deviating prisms. In contrast, right-deviating prisms decreased the efficiency of voluntary attention in both left and right space. The experiment performed by Morris et al. [51] was less conclusive in the sense that neither adaptation to left-deviating prism nor adaptation to right-deviating prism significantly modified a visual search task. However, results presented in this latter article showed a clear decrease of reaction time and percentage of error in the left visual space (not present in the right visual space) after left-deviating prism. Altogether, the data available in the literature suggest that the cognitive effects of prism adaptation in healthy subjects depend on the direction of the optical shift. The present results suggest that the same is true for unilateral neglect patients, but in the opposite direction. Spatial neglect is improved only by adaptation to rightward optical shifts and spatial cognitive functions tested on healthy subjects are affected mainly after adaptation to a left-ward shift. This coherence allows proposing an integrated model of the effects of prism adaptation on spatial cognition, whereby the lateralized effects of adaptation on the cerebellum would affect the controlateral hemisphere [52].

### 4.3. Neural Mechanisms Underlying Prism Adaptation Beneficial Effect on Left Spatial Neglect

The neural substrate underlying the therapeutic effect of this method remains to be fully elucidated. Our study was not specifically designed to deal with this issue but argues at least for an initial lateralized bottom-up activation implicated in the detection of the right visual error during the first pointing movements through prisms. In a recent functional imaging study performed on healthy subjects, we used event-related fMRI to analyze dynamic changes in brain activity during a prolonged exposure to visual prisms [53]. Results suggest that during exposure to a leftward prismatic deviation, error-detection was processed in the left intraparietal sulcus, errorcorrection involved the left parietooccipital sulcus, and visuomotor realignment implicated the right cerebellum. Furthermore, the activation observed bilaterally in the superior temporal cortex during the late phase of prism exposure was thought to mediate the effects of prism adaptation on cognitive spatial representations.

The mechanism by which such lateralized sensori-motor plasticity induced by prism adaptation can improve spatial neglect remains unclear. Moreover, the gap might be important between what we know about sensori-motor plasticity in normal subjects and what happens in brain-damaged neglect patients.

In a functional imaging PET study, we investigated the anatomical substrates underlying the beneficial effect of prism adaptation in five patients with left spatial neglect following right stroke [54]. We used a covariation analysis to examine linear changes over sessions as a function of neglect improvement. This study confirmed that a low-level sensori-motor adaptation can modulate several cortical areas involved in spatial cognition and gives further support to a bottom-up mechanism. Altogether, the following model is proposed: error signals induced by prisms are initially processed by the left occipitoparietal cortex, then forwarded to the right cerebellum where visuomotor realignment takes place. The clinical benefit would result from the modulation of left-hemisphere areas via a bottom-up signal produced by the cerebellum. These areas would partially match those involved in spatial cognition in the right hemisphere, and their modulation would improve interhemispheric rebalancing. The basic idea proposed here is that the activation of the right cerebellum by prism adaptation would play a negative influence on the activation of the left cerebral hemisphere. A recent support for such interaction was provided by Pope and Miall [55] who explored the effect of cerebellar activity modulation on cognitive tasks. One classic idea about the contribution of the cerebellum to cognitive function has been that the processing capacities developed in the cerebellum for sensorimotor control could also turn out to be useful for cognitive operations. Accordingly, the expectation is that reducing cerebellar activity on one side would impair contralateral hemispheric functions. However Pope and Miall revealed that cathodal tDCS on the right cerebellum resulted in an improvement of several cognitive tasks known to rely on the left cerebral hemisphere functions. The reciprocal arguments that enhancing right cerebellum activity by prism adaptation may inhibit left cerebral cortex function and that downregulating right cerebellum activity by cathodal tDCS may enhance left cerebral cortex function provide a general coherence to the idea that cerebellocortical interactions contribute to the expansion of prism adaptation effects to cognitive functions.

## Figures and Tables

**Figure 2:**
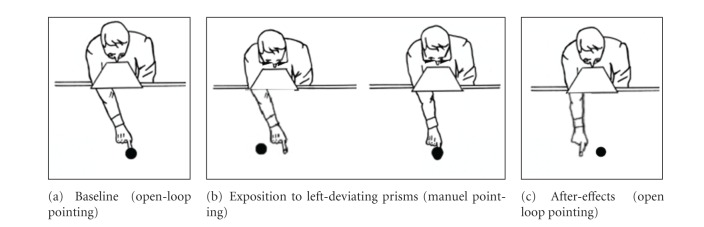
Left-deviating prism adaptation schematic procedure.

**Table 1:** Clinical profiles of each patient.

Patients number	1	2	3	4	5
Sex	M	F	F	F	F
Age	80	75	73	67	74
Time after onset (mt)	2	1.5	2.5	1	1
Motor deficit	L hemiparesis	L hemiplegia	L hemiplegia (transient)	L hemiplegia	L hemiparesis
Somatosensory deficit	+	+	−	+	+
Hemianopia	+	−	+	−	−
Constructive apraxia	−	+	−	−	+
Type of lesion	I (MCA)	H	I (MCA)	I (MCA)	I (MCA)

Motor and somato-sensory deficits were assessed by a classical clinical examination. Presence of hemianopia was assessed by means of the Goldman perimetry. Constructive apraxia was assessed on copying geometrical drawings.

Abbreviations—+: present; −: absent; mt: month; F: female; M: male; L: left, H: hemorrhagic; I: ischemic; MCA: middle cerebral artery.
